# Schizophrenia treatment with a combination of two LAI antipsychotics: A case report

**DOI:** 10.3389/fpsyt.2022.975531

**Published:** 2022-09-16

**Authors:** Marcin Jarosz, Karina Badura-Brzoza

**Affiliations:** Department of Psychiatry, Faculty of Medical Sciences in Zabrze, Medical University of Silesia in Katowice, Katowice, Poland

**Keywords:** antipsychotics, treatment, long-acting injectable, polytherapy, treatment-resistant schizophrenia, non-compliance

## Abstract

Preventing the relapse of a psychotic episode is a challenge for the treatment of schizophrenia. Patients with schizophrenia suffer from a few to a dozen relapses in their lifetime. The use of long-acting injectable (LAI) antipsychotics in the treatment of schizophrenia is associated with less frequent recurrences of psychotic symptoms, better compliance, and better quality of life. The aim of the report is to present the findings of the successful management of treatment-resistant schizophrenia in a patient with persistent non-compliance using a combination of typical and atypical LAI antipsychotics. Since there was a history of non-adherence (irregular controls in outpatient clinics) by the patient, clozapine was not considered a therapeutic option. At the start of the treatment, olanzapine LAI was administered to the patient at a dosage of 300 mg fortnightly because of the good response and tolerance reported in the previous treatment. The treatment was continued for several weeks, and because of the persistence of constant delusions, labile affect, and aggressive behavioral tendencies, a second antipsychotic, zuclopenthyxole, was added, which was initially administered orally. After 4 weeks of combined treatment, the patient's mental state improved. There was no report of delusions, and his mood was much more stable. Zuclopenthyxole was switched to the LAI antipsychotic form due to the patient's history of persistent non-compliance, lack of insight into the disease, and the risk of aggressiveness toward others. Then, 200 mg of zuclopenthyxole decanoate was administered fortnightly. The patient was discharged from the hospital without any symptoms of delusions or hallucinations. The patient's clinical state presented negative symptoms, of which avolition and diminished social activity were dominant. The patient tolerated the treatment well, and sedation and extrapyramidal symptoms were not observed. The patient continued the injections alternately (one injection per week) to obtain regular visits to the outpatient clinic.

## Introduction

Prevention of a relapse of a psychotic episode in the treatment of schizophrenia is a challenge. Patients living with schizophrenia suffer from a few to a dozen relapses in their lifetime. Each relapse carries the risk of worsening the long-term prognosis of the disease and affects the patient's quality of life. The life expectancy of patients with schizophrenia is shorter by 15–20 years (1). Untreated psychosis and short time remission are associated with higher mortality (2), and inadequate treatment might be connected with aggressive behaviors (3). The use of antipsychotics in the treatment of schizophrenia is associated with 40% lower mortality. The risk of death is 30% lower in the group of patients who are treated with long-acting injectable (LAI) antipsychotics compared to oral antipsychotics, which is due to their neuroprotective activity (4). The first LAI, fluphenazine enanthate, was developed in 1966. Although the concept of LAIs was not initially accepted by the medical profession, many subsequent studies proved to have lower relapse rates of the disease when using LAIs in treatment. Fluphenazine enanthate was administered every 2 weeks. Almost 18 months later, fluphenazine decanoate was added to the treatment, and it was administered every 3 weeks (5). The introduction of oral second-generation antipsychotics (SGA) was supposed to be associated with better adherence rates. However, these medicines did not improve the treatment compliance of the patient. In 2001, long-acting risperidone was the first LAI SGA (6).

Antipsychotic polytherapy is used in up to 30% of patients (7) who do not respond to monotherapy. Tiihonen et al. showed that polytherapy was associated with a lower risk of rehospitalization (8). Therefore, certain types of polypharmacy may be feasible in the treatment of schizophrenia. One of the management options in treatment-resistant schizophrenia (TRS) or persistent non-compliance can be a combination of two LAI antipsychotics. TRS is defined as a history of at least two antipsychotic trials (one of them an atypical antipsychotic) with adequate doses administered at a 4–6 week period, without satisfactory responses, particularly in terms of persistent psychotic symptoms (9). The aim of this case report is to present the successful management of treatment-resistant schizophrenia in a persistently non-compliant patient using a combination of typical and atypical LAI antipsychotics.

## Case report

The patient was a 34-year-old single man. He was raised by both parents, and there was no family history of mental illness. The patient had secondary education, and he was employed in Zinc Works until 2010. There was no history of alcohol or drug use. He has been treated since 2009 with a diagnosis of schizophrenia. The course of the disease was unstable, and exacerbations were mainly caused by the irregular intake of medications. Because of this fact, he was hospitalized many times, and his hospital stay lasted up to several months. The main reasons for repeated hospitalizations were disorderly behavior and aggression.

The patient was first hospitalized in a psychiatric unit in 2009 for aggressive behavior; he was aggressive with his parents and had attacked a paramedic. The patient claimed to be a trained special agent. The first symptoms were observed by his family 2 years prior, and he had become withdrawn and had broken off all contact with his friends. He used offensive language in contact with family. Perphenazine enanthate was used in his treatment, and the patient responded well to the therapy. His second hospitalization was in 2011 under similar circumstances. Treatment was attempted with risperidone and amisulpride, but there was no response. Finally, improvement was obtained with the use of quetiapine. His third hospitalization took place in January 2021. The patient received olanzapine LAI and valproic acid. However, after being discharged from the hospital, he discontinued his injections.

On 13 March 2021, he was admitted to the psychiatric department with a history of attacking people in the street, for which he was apprehended by the police. In the physical examination, obesity (BMI, 30.5 kg/m^2^) and cutaneous purulent changes in the armpit area were noted, but no significant abnormalities were observed. The patient's blood pressure and heart ratio were normal. There were no abnormalities in the neurological examination. The patient negated somatic diseases except for acne inversa. In the patient's mental state examination at admission, he was noted to be conscious, oriented in time, place, and person, with normal psychomotor activity. He demonstrated signs of blunted affect, and he reported passivity experiences, saying that others could manipulate drug levels in his blood with radio waves, thus changing his moods. Additionally, he reported delusions of reference. Other abnormalities of thought included loose associations and paralogical thinking: He said that he could not be arrested because “if someone is detained illegally the building will collapse” and “This is the theory of construction.” Furthermore, the patient denied attacking another person. Hallucinations or suicidal ideations were not observed. He scored 90 points on the Positive and Negative Syndrome Scale (PANSS) (Table 1). There were no abnormalities in blood tests, except for hypercholesterolemia and hypertriglyceridemia. Considering the long-term course of the disease, frequent recurrences of episodes with acute psychotic symptoms and significant behavioral disturbances, including behaviors potentially dangerous to others, it would seem that clozapine could be the drug of choice in such a situation.

**Table 1:** The PANSS score before and after the intervention with two LAIs.

**Symptoms**	**Score before the intervention**	**Score after the intervention**
P1 Delusions	7	1
P2 Conceptual disorganization	5	3
P3 Hallucinatory behavior	2	1
P4 Excitement	3	1
P5 Grandiosity	3	1
P6 Suspiciousness/persecution	3	1
P7 Hostility	3	1
N1 Blunted affect	3	3
N2 Emotional withdrawal	5	5
N3 Poor rapport	4	4
N4 Passive/apathetic social withdrawal	5	5
N5 Difficulty in abstract thinking	4	4
N6 Lack of spontaneity and flow of conversation	2	3
N7 Stereotyped thinking	3	2
G1 Somatic concern	1	1
G2 Anxiety	1	1
G3 Guilt feelings	1	1
G4 Tension	1	1
G5 Manierisms and posturng	1	1
G6 Depression	1	1
G7 Motor retardation	1	1
G8 Uncooperativeness	2	1
G9 Unusual thought content	5	2
G10 Disorientation	1	1
G11 Poor attention	2	2
G12 Lack of judgment and insight	5	2
G13 Disturbance of volition	2	1
G14 Poor impulse control	5	1
G15 Preoccupation	3	2
G16 Active social avoidance	3	3
Total	90	57

However, clozapine was finally not considered a therapeutic option because of non-adherence (irregular controls in outpatient clinics) in the patient's previous history. So, the patient was treated with 300 mg of olanzapine LAI fortnightly due to good response history and tolerance to such treatment._In addition, the patient received diazepam orally at 15 mg doses/day and haloperidol orally at 15 mg doses/day. This treatment continued for several weeks, and a second antipsychotic (zuclopenthyxole) was added because of the persistence of constant delusions, labile affect, and aggressive behavior tendencies. Valproic acid was not considered due to his obesity. Initially, zuclopenthyxole was administered orally at a dosage of 75 mg/day. After 4 weeks of combined treatment, his mental state improved. There was no report of delusions, the mood of the patient was much more stable, and awareness of his symptoms was better. A temporary increase in aspartate transaminase (40, 8 U/L) and alanine transaminase (67, 2 U/L) was observed. Other blood tests and ECG reports came up with normal volumes. Zuclopenthyxole was switched to a depot form due to persistent non-compliance in previous treatment, poor insights, and the risk of being aggressive to other people. Zuclopenthyxole decanoate in 200 mg doses was administered fortnightly. The patient was discharged from the hospital on the 70th day of hospitalization. In the examination of his mental state, he was noted to be conscious and oriented in time, place, and person, with normal psychomotor activity. Blunted affect was reported. He did not report any delusions or hallucinations and did not present suicidal ideations. The patient's clinical state presented negative symptoms, of which avolition and diminished social activity were dominant. The total score was 57 points on the PANSS (Table 1). No significant abnormalities in blood tests or ECG were found. The patient tolerated the treatment well. Sedation and extrapyramidal symptoms were not observed. The patient continued taking the injections alternately (one injection per week) to obtain regular visits to the outpatient clinic. The patient received rosuvastatin at a dose of 20 mg/day and dietary recommendations (reduction of monosaccharides and animal fats) due to dyslipidemia. There were attempts to introduce individual and family therapy; unfortunately, they constantly failed. Due to the disturbances in the structure of thinking with its distinct rigidity, the therapy and psychoeducation attempts did not bring any results. Additionally, all attempts at rehabilitation procedures offered by social help were rejected by the patient. Currently, the patient remains under the care of a psychiatric outpatient clinic outside our unit; therefore, further history is unknown.

## Discussion

There are few reports in the literature on the use of combined LAI antipsychotics in treatment. This case report presents the treatment of a patient with a severe and unstable course of the disease accompanied by a lack of cooperation, which resulted in aggressive behavior toward other people. The previous treatment administered to this patient seemed to be insufficient, leading to incomplete remission of symptoms, and above all, it did not alleviate dysphoria and did not improve the patient's insight. Incomplete remission and the patient's negative attitude toward the treatment resulted in permanent discontinuation of medicines, which in turn led to a rapid deterioration of the mental state and subsequent aggressive behaviors. In the context of the above information, the patient started to be treated through a combination of two LAI antipsychotics and obtained significant clinical benefits. The combined LAI therapy was safe and well-tolerated. Better tolerance can result from the more predictable pharmacokinetics of LAI antipsychotics compared to the oral form. Side effects related to the daily peak concentration may be eliminated (10).

As a rule, the calculation of total doses for oral vs. LAI antipsychotics will usually show lower doses for the LAI (6). The data imply that there are no significant differences between LAI and oral antipsychotics regarding the risk of death (excluding suicide and accident), any extrapyramidal side effects, QTc average change, and abnormality in blood tests (11).

The basic clinical indications for LAI antipsychotic treatment in schizophrenia are as follows: unsatisfactory clinical improvement, unsatisfactory functioning improvement, and high risk of recurrence (among others: rehospitalization during the period of <12 months, interruption of oral pharmacotherapy lasting more than 2 weeks) (12). Treatment guidelines do not address combining two LAI antipsychotics. Hence, when deciding whether to use two neuroleptics at the same time, it is important to make decisions with a team of doctors, closely monitor the patient's condition, and conduct thorough documentation. When a patient does not adhere to required laboratory monitoring, clozapine cannot be an option in the management of treatment-resistant schizophrenia. In this case, it is possible to consider the combination of two LAI antipsychotics (13). In the literature, only a few case reports can be found detailing the use of combined application of typical and atypical LAI antipsychotics (14–16). In all cases, significant improvement and no adverse effects were reported.

McInnis et al. used a combination of two LAI antipsychotics in three cases of adolescents with severe psychosis and aggression (17). The research showed that the combination of LAI antipsychotics may be an effective and safe therapeutic option in adolescents.

In another case series, Fang-Ling et al. described the successful management of treatment-resistant schizophrenia in two patients. They used typical and atypical LAI antipsychotics in an alternating administration sequence. Both patients tolerated this treatment well (18).

Another case report demonstrated that the safety of combined LAI antipsychotic treatment was about the use of three LAI antipsychotics in a patient with a psychotic disorder in the course of Huntington's disease. The patient received haloperidol at a dose of 100 mg, i.m., every 7 days with simultaneous administration of risperidone at a dose of 50 mg, i.m., every 14 days and olanzapine at a dose of 405 mg, i.m., every 1 month. Side effects after administration of LAI antipsychotics were not observed (19). It seems that the use of two long-acting neuroleptics may be helpful in the treatment of drug-resistant, non-cooperative patients who present a risk of aggressive and dangerous behaviors.

## Conclusion

Our case report and other cited cases show that the combination of two (or even three) LAIs can possibly be a safe therapeutic option. It seems to be an option in patients with persistent non-compliance, where clozapine is not a viable therapeutic option. Unfortunately, at this moment, there are no studies detailing the safety of the combination LAI treatment. This is an area for further research.

## Data availability statement

The original contributions presented in the study are included in the article/supplementary material, further inquiries can be directed to the corresponding author/s.

## Ethics statement

Ethical review and approval was not required for the study on human participants in accordance with the local legislation and institutional requirements. The patients/participants provided their written informed consent to participate in this study. Written informed consent was obtained from the individual(s) for the publication of any potentially identifiable images or data included in this article.

## Author contributions

MJ wrote the case report and article. KB-B reviewed the case report and article as senior author. Both authors contributed to the article and approved the submitted version.

## Conflict of interest

The authors declare that the research was conducted in the absence of any commercial or financial relationships that could be construed as a potential conflict of interest.

## Publisher's note

All claims expressed in this article are solely those of the authors and do not necessarily represent those of their affiliated organizations, or those of the publisher, the editors and the reviewers. Any product that may be evaluated in this article, or claim that may be made by its manufacturer, is not guaranteed or endorsed by the publisher.
